# Natural killer cells in intracranial neoplasms: presence and therapeutic efficacy against brain tumours

**DOI:** 10.1007/s11060-013-1265-5

**Published:** 2013-10-02

**Authors:** Justyna Kmiecik, Jacques Zimmer, Martha Chekenya

**Affiliations:** 1 Department of Biomedicine, University of Bergen, Jonas Lies vei 91, 5009 Bergen, Norway; 2 Laboratoire d’Immunogénétique-Allergologie, CRP-Santé, Luxembourg, Luxembourg

**Keywords:** Tumour infiltrating lymphocytes, NK cells, Brain tumour, Prognosis, Immunotherapy

## Abstract

Natural killer (NK) cells are lymphocytes that play an important role in anti-tumour immunity. Their potential against brain cancer has been demonstrated in vitro and in vivo, both as a direct anti-tumour agent and in experimental therapies stimulating endogenous NK cell cytotoxicity. However, the clinical translation of these promising results requires detailed knowledge about the immune status of brain tumour patients, with focus on the NK cell population. In this report, we provide an overview of the studies investigating NK cell infiltration into the tumour, emphasizing the need of revision of the methodologies and further research in this field. We also discuss the potential of using autologous or allogeneic NK cells as effector cells in cellular therapy against brain cancer and developing immunotherapies stimulating endogenous NK cell-mediated anti-tumour response, such as blocking inhibitory killer immunoglobulin-like receptors. Combination of NK cell adoptive transfer with targeted therapies, such as anti-EGFR therapeutic antibody (CetuximAb) could also be a potent strategy.

## Introduction: why focus on natural killer cells for brain tumours?

Brain tumours are intracranial neoplasms located in the brain and meninges (excluding haematopoietic malignancies occurring in the brain) and they account for approximately 1.4 % of all cancers [1, 2]. The incidence of brain and central nervous system tumours worldwide in 2008 was 237,913 new reported cases and is projected to increase to 357,377 by 2030 [3]. Depending on the grade of malignancy, the current standard treatment includes surgery, chemo- and/or radiotherapy. Despite advances in anti-tumour therapy, patients’ outcomes remain poor due to inevitable recurrence and malignant progression. One of the challenges in treating brain tumours is the presence of the blood brain barrier (BBB) that limits the access of systemically administered drugs into the tumour site. Due to the BBB, lack of lymphatic drainage and lack of professional antigen presenting cells, brain tumours have been also considered protected from immune system surveillance. However, this concept has been revised, as immune cells have been shown to infiltrate brain tumours [4] and play important roles in both anti-tumour immunity [4] and tumour progression [5, 6].

Natural killer (NK) cells are large granular lymphocytes of the innate immune system. These cells are able to directly lyse infected or transformed cells without specific immunization [7]. NK cells can also recognise the Fc part of antibodies via low affinity FcγRIIIA (CD16) receptor and perform antibody dependent cellular cytotoxicity (ADCC) of antibody-coated cells [8]. Moreover, they secrete various cytokines and chemokines, such as interferon gamma (IFN-γ) [7]. The recognition of target cells and activation of NK cells is controlled by a balance between activating and inhibitory signals mediated by binding of the ligands expressed on target cells to the receptors expressed on NK cells [9, 10]. The inhibitory receptors include NKG2A [11] and inhibitory killer immunoglobulin-like receptors (KIRs) [12, 13] that recognise major histocompatibility complex (MHC) class I molecules, which are often down regulated on virus-infected or tumour cells. The activating signals are mediated by natural cytotoxicity receptors (NCRs) [14] and NKG2D [14] that recognise ligands expressed on infected or transformed cells, but absent on most normal cells. Some KIR receptors also mediate activating signals, however, they engage the MHC class I ligands with lower affinity than inhibitory KIRs [15]. Upon activation, NK cells induce apoptosis of target cells by secreting granules containing perforin and granzymes [16] or by signalling via death receptors [17].

NK cells play an important role in anti-tumour immunity as several lines of evidence link NK cell surveillance [18], anti-tumour activity [19] and prognostic significance [20–24] in various types of cancers. The potential of NK cells as effectors against brain tumours has been demonstrated in vitro [25, 26] and in vivo [27, 28, 29]. However, translating these promising results into the clinic requires answers to several important questions related to patients’ immune status: (1) Do NK cells infiltrate brain tumours and if yes, are they functional? (2) What is the prognostic significance of NK cells’ activity and their infiltration into the tumour? (3) How do the tumour cells escape NK cell-mediated surveillance and killing? Clinical trials using NK cells against brain tumours have already been conducted [30] and some are in progress or recently completed (for example clinicaltrials.gov: NCT00823524 and NCT00909558). Moreover, other experimental therapies might be influenced by patients’ NK cell activity [31] and also several treatment strategies have been shown to stimulate NK cell-mediated lysis of tumour cells [8, 27, 28, 32]. Therefore, it is of great importance to take into consideration the brain tumour patients’ immune status and encourage further research in this direction. Our review summarizes what is already known in this field and what aspects need further investigation. We also provide an overview of pre-clinical studies conducted and discuss the clinical application of NK cell-based therapy.

## What is the immune status of brain tumour patients?

Numerous studies have investigated the immune system of brain tumour patients, mainly in the context of malignant glioma. Brain tumour patients suffer from extensive immunosuppression due to lymphopenia, decreased lymphocyte proliferation [33, 34] and diminished cytotoxic activity [35], reduced MHC class I expression on monocytes [33, 34] and predominance of anti-inflammatory T-helper 2-type (Th2) cytokine production [33]. In one of the first studies, Servadei et. al analysed cytotoxic functionality of peripheral blood lymphocytes isolated from anaplastic glioma patients and compared with those from bladder and kidney cancer patients and healthy donors [35]. In all three types of cancers they observed decreased ADCC and decreased spontaneous lymphocyte mediated cytotoxicity (SLMC) when comparing to healthy donors, to the greatest extent in anaplastic glioma patients. Reports investigating NK cell numbers and activity in the peripheral blood of glioma patients show conflicting results (reviewed in Dix et al. [33]. The functionality of circulating NK cells is often affected in brain tumour patients and it may result from immunosuppressive factors released by tumour cells. An example is transforming growth factor-beta (TGFβ) that down-regulates the expression of NKG2D activating receptor on NK cells isolated from glioblastoma patients compared to those from meningioma patients [36]. Another important factor to be considered is the influence of the standard treatment. Brain tumour patients are immune-compromised due to treatment with steroids [37]. Moreover, decreased absolute numbers of NK cells have been observed in the blood of glioblastoma patients receiving concomitant radiation therapy and temozolomide™ [38].

## Do NK cells infiltrate the brain tumour?

The remaining question was whether the NK cells infiltrate the tumour site. In 1988, Stevens et al. characterised the immune infiltrates in gliomas, carcinoma metastases, craniopharyngiomas and meningiomas [39]. To identify NK cells within the brain tumour tissue, the immunohistochemistry (IHC) technique with Leu7 (CD57) and Leu11b (CD16) antibodies was used (Table 1). They observed that in most glioma cases NK cells were absent, while carcinoma metastases and craniopharyngiomas were more frequently infiltrated by NK cells. Single NK cells were detected in some meningiomas. In all cases, NK cells constituted a minor fraction among all immune cells infiltrating the tumour, leading the authors to conclude that NK cells do not play an important role in anti-tumour immunity in brain cancer. Similar results were obtained by Vaquero et al. for brain metastases [40] with use of the IOT-10 antibody that also recognises CD57. In addition, they showed that the degree of NK cell infiltration varied depending on the origin of metastasis. In a recently published work, the same group reported no correlation of the degree of CD57+ NK cells with the clinical outcome of patients with brain metastasis [41]. The critical point of all those studies is that the CD57 marker is expressed only on a subset of NK cells and is also expressed on a significant fraction of T cells [42]. Therefore a more precise analysis is needed to determine the true frequency and degree of NK cell infiltration in brain tumours.

**Table 1:** NK cell infiltration in intracranial tumours: summary of reviewed literature

Authors	Tumour type	Method, antibodies	Degree of NK cell infiltration	Distribution of NK cells in the tumour tissue
Stevens et al. [39]	Gliomas grade IV	IHC, Leu7 and Leu11b	Low	Within perivascular cuffs
	Gliomas grade I–II		No infiltration	
	Carcinoma metastases		Intermediate	Around blood vessels and within tumour parenchyma
	Craniopharyngiomas		Intense	No specific distribution
	Meningiomas		Low	–
	Other tumours		Low	–
Vaquero et al. [40]	Brain metastases	IHC, IOT-10	Less than 10 % of TILs in most cases (39/40)	Mainly associated with vessels and stroma
Yang et al. [43]	Glioblastomas	IHC, CD56	Perivascular: intermediate or extensive in app.70 % cases; Intratumoural: intermediate in app. 25 % cases, none in app. 50 % cases	Mostly perivascular/extratumoural
	Pilocytic astrocytoma		No infiltration	
Rossi et al. [46]	Oligodendrogliomas	IHC, Leu11b	No infiltration	
Domingues et al. [47]	Meningiomas	Flow cytometry, NK cell population defined as CD3-CD19-CD56+	0.2 ± 0.3 % of all cells	

*IHC* immunohistochemistry, *TIL* tumour-infiltrating lymphocytes, – no data reported

Contrasting results were reported by Yang et al. [43] in the most recently published work investigating immune cell infiltrates in glioblastomas and comparing it with pilocytic astrocytomas. According to their report, NK cells frequently infiltrated the glioblastomas, whereas this infiltration was negligible in pilocytic astrocytomas. Therefore, contrary to Stevens et al. [39], the authors concluded that NK cells may play an important role in anti-tumour immune responses in glioblastoma patients. In this study the NK cells were identified as CD56+ cells. This method alone might give inaccurate results as CD56 is also expressed by some cytotoxic T cells [44]. According to our observations, NK cells were one of the least numerous immune cell populations of all tumour infiltrating immune cells in glioblastomas (2.11 % ± 0.54, mean ± SEM) and were predominantly CD56^dim^CD16^−^ [45]. These results are based on multicolour flow cytometric phenotyping of patients’ glioblastoma (GBM) tumour biopsies. The NK cell population was defined as CD3 negative CD56 positive.

Concerning oligodendrogliomas, Rossi et al. reported absence of NK cells in those tumours [46]. However, they evaluated the expression of Leu11b (CD16) that is expressed only on the major subpopulation of NK cells. In the light of the observations made by Stevens et. al [39], who did not detect Leu11b positive cells despite the presence of Leu7 (CD56) positive infiltrates in various brain tumour specimens and our own studies that did not detect CD16 positive NK cells in GBMs, it is possible that in oligodendrogliomas a similar expression pattern of CD56^+^CD16^−^ occurs as in the other gliomas.

The most recent study focusing on meningiomas was conducted by Domingues et al. [47] and demonstrated the presence of NK cells within the tumour. Similar to our results and those of Stevens et al., [39], NK cells were one of the least numerous immune cell populations infiltrating the tumour.

## Are NK cells a potent anti-tumour agent against brain cancer? Functional studies

A number of in vitro and in vivo functional studies have been performed in order to investigate the role of NK cells in anti-tumour immunity in brain cancers and the potential of using them as a therapeutic agent. Alizadeh et al. [28] investigated the therapeutic efficacy of a toll-like receptor 9 (TLR9) ligand, CpG-oligodeoxynucleotides (CpG-ODN), in vivo in a murine glioma model. They showed that NK cell numbers in brain, blood and spleen decreased with tumour growth, possibly as a result of tumour-induced immunosuppression. However, they also demonstrated that the therapy they used against glioma induced host immune responses and NK cells mediated the resistance to tumour re-challenge. Another group demonstrated increased cytotoxic activity of splenic NK cells isolated from glioblastoma-bearing animals treated with recombinant adeno-associated virus 2 encoding IL-12 [48]. Dendritic cell (DC) vaccination has also been shown to stimulate IFNγ secretion by NK cells and increase their number in the peripheral blood in GBM patients [49]. On the other hand, Alvarez-Breckenridge et al. showed in vivo, that NK cells can negatively influence virotherapy against glioblastoma [31]. Castriconi et al. [25] evaluated the susceptibility of the glioblastoma stem-like cells to NK cell-mediated lysis in vitro. They found that both allogeneic and autologous activated NK cells were able to efficiently kill the GBM cells. However, the GBM cells were resistant to resting NK cells. Avril et al. [26] compared the GBM cells cultured under serum-free conditions with those serum-cultured in a series of cytotoxicity assays using activated NK cells and T cells as effectors. They reported that GBM stem-like cells were more susceptible to both NK cell- and T cell-mediated lysis. Moreover, they showed that in combination with the therapeutic antibody cetuximab, NK cells were able to kill GBM stem-like cells via ADCC. Our team recently demonstrated that combination treatment with NK cells +mAb9.2.27 against the NG2/CSPG4 proteoglycan diminished tumor growth that was associated with reduced tumor proliferation, increased cellular apoptosis and prolonged survival compared to vehicle and monotherapy controls. Therapeutic ADCC was mediated by recruitment of CCR2low macrophages into the tumor microenvironment, increased ED1 and MHC class II expression on microglia that rendered them competent for GBM antigen presentation, as well as elevated IFN-γ and TNF-α levels in the cerebrospinal fluid compared to controls [29]. The advantages and relevance of these studies were the use of purified NK cells and patient-derived GBM cells.

## Can the anti-tumour potential of NK cells be exploited in the clinic?

The prognostic significance of NK cells’ activity has been demonstrated in patients with various solid tumours [20–22, 24]. A higher level of CD57 positive cells infiltration into the tumour correlates with better survival of patients with oesophageal squamous cell carcinoma [20], squamous cell lung cancer [24] and gastric carcinoma [21]. As mentioned before, CD57 is expressed also on a subset of T cells [42], therefore very likely both NK cells and T cells contribute to better patient outcomes. In the study of Kondo et al., a high activity of peripheral NK cells positively correlated with longer survival of colon cancer patients [22]. They also observed that cumulative 5-year metastasis-free rates were higher in the group of patients with high NK cell infiltration (90 vs. 60 %). These data suggest NK cells play an important role in controlling metastases. Moreover, tumour cells have been shown to express ligands for activating receptors expressed on NK cells [50, 51], making them potentially susceptible to NK cell-mediated lysis. Various immunotherapies against brain cancer have been tested so far (reviewed in [52]), including DC vaccination, adoptive T cell transfer, and transfer of lymphokine activated killer (LAK) cells. Most of the clinical trials referring to NK cell activity utilized autologous LAK cells combined with IL-2 injections (recently reviewed in [53]. LAK cells are obtained from peripheral blood mononuclear cells (PBMCs) cultured at conditions that stimulate their expansion and activation. The final product is a mixture of T cells (majority of them are cytotoxic T lymphocytes (CTLs) and NK cells. Those trials demonstrated partial efficacy and moderate adverse effects (mostly edema) [54, 55]. The weak point of those studies is that the effector cell populations contributing to anti-glioma effect have not been characterised and the mechanisms of treatments tested have not been investigated. However, they showed the potential of cellular therapy against the most malignant brain tumour.

**Fig. 1:**
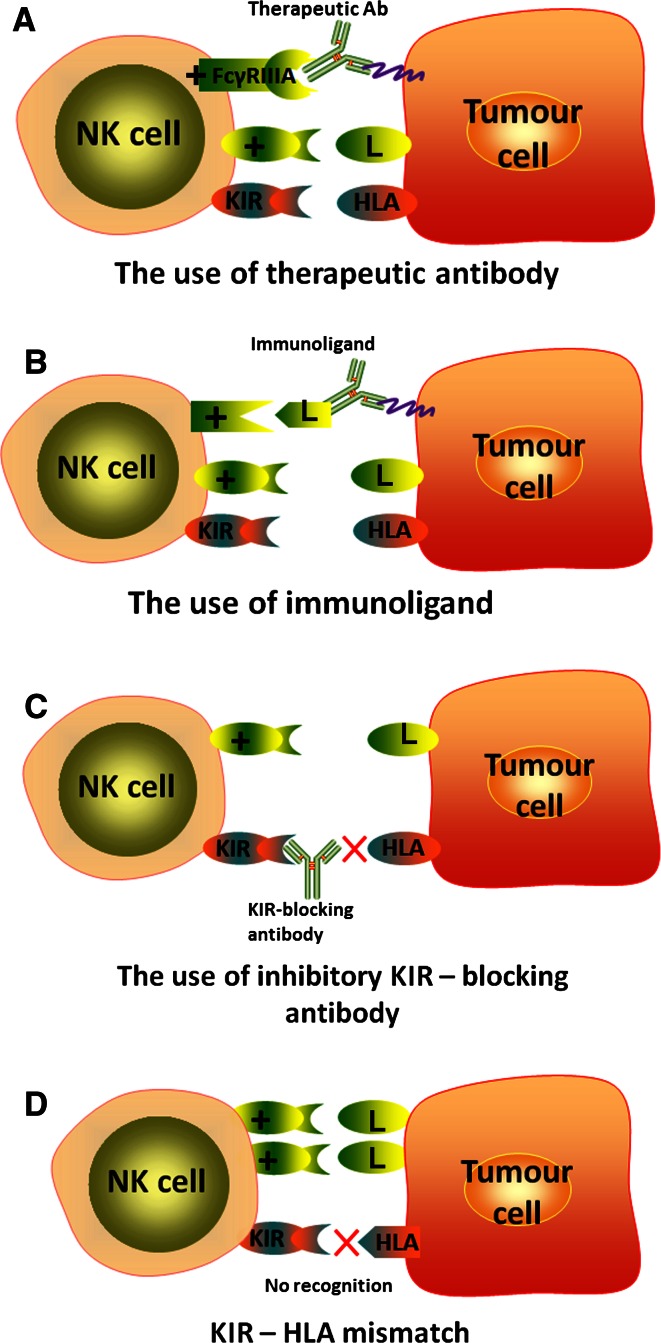
Treatment strategies for using NK cells against brain tumours. **a** Therapeutic antibodies, such as cetuximab (anti-EGFR antibody) can induce antibody-dependent cellular cytotoxicity (ADCC) mediated by FcγRIIIA receptor (CD16) expressed on NK cells. **b** Immunoligand is a fusion protein: the part recognising specific antigen is conjugated to ligand for activating receptor. Immunoligand binds tumour-specific antigen and stimulates NK cells (endogenous and/or transferred, both autologous and allogeneic) via interaction with activating receptor (e.g. NKG2D). **c** Applying KIR-blocking antibody reduces the inhibition of both endogenous NK cells as well as adoptively transferred autologous NK cells. **d** Transfer of allogeneic NK cells with KIR-HLA mismatch approach. Lack of recognition of tumour’s HLA by inhibitory KIR receptors results in the absence of inhibitory signals and NK cell activation. + activating receptor, *L* ligand

**Fig. 2:**
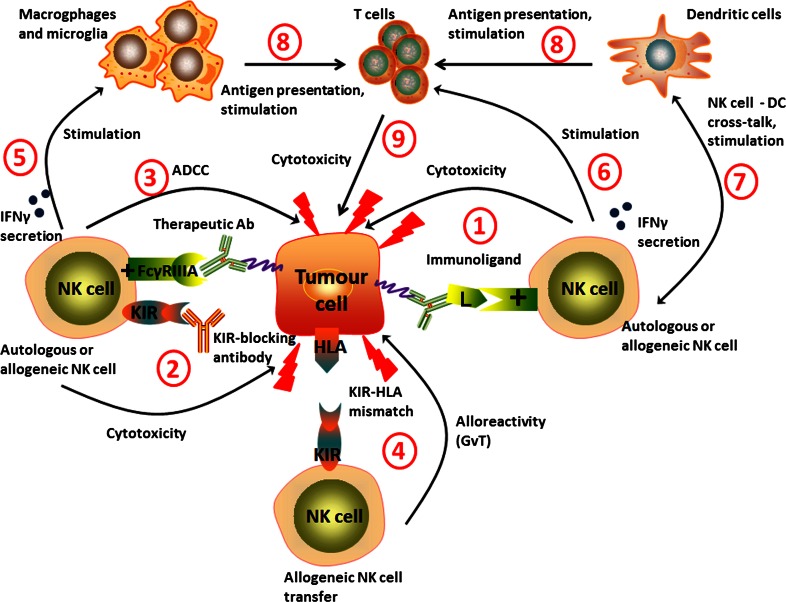
The interplay of NK and other immune cells in anti-tumour responses. Endogenous and/or adoptively transferred autologous NK cells can be stimulated by immunoligands (*1*) and/or KIR blocking antibodies (*2*). Therapeutic antibodies such as cetuximab (anti-EGFR antibody) can induce antibody-dependent cellular cytotoxicity (ADCC) (*3*). Other possible strategy is cellular therapy with allogeneic NK cells with KIR-HLA mismatch approach (graft vs. tumour effect, GvT) (*4*). Stimulated and/or alloreactive NK cells are able to directly kill the tumour cells, as well as to secrete pro-inflammatory cytokines such as IFNγ to further stimulate other immune cells: macrophages, microglia (*5*) and T cells (*6*). NK cells may play an important role in DC vaccination due to DC-NK cell cross-talk further stimulating anti-tumour immune response (*7*). Stimulated macrophages, microglia and DC can present tumour-associated antigens (*8*) and induce CTLs-mediated cytotoxicity (*9*). + activating receptor, *L*: ligand

Therefore, using pure NK cells against solid tumours becomes an attractive alternative strategy to using LAK cells. To date, only one phase I clinical trial has been conducted, where pure autologous NK cells were used as effector cells against brain tumours [30]. In this study, 9 patients with recurrent malignant glioma received intracranial or intravenous injection of NK cells expanded from patients’ PBMCs. In 4 of 9 patients, the tumour regression was recorded as magnetic resonance imaging (MRI) radiological response. The benefit in terms of survival outcomes was not evaluated. The authors concluded their therapy was less toxic but was as efficient as LAK cells combined with IL-2. The lower toxicity might be due to the considerably lower doses of IL-2 used. The limited efficacy of experimental therapies utilizing autologous LAK or NK cells might be due to the local and highly integrated tumour-induced immune suppression and immune escape mechanisms. CTLs require antigen presentation and co-stimulation signals that are often impaired in brain tumour patients [45]. Our group has also demonstrated that GBM cells highly express MHC class I molecules [45], that are ligands for inhibitory receptors expressed on NK cells [12]. Therefore, engaging NK cells to kill the tumour cells might require additional, combination treatments that will enhance their activity and/or overcome tumour immune escape mechanisms. One possibility is the use of tumour specific antibodies that would induce ADCC mediated by engaging the FcγRIIIA receptor (CD16) on NK cells (Fig. 1a). An interesting approach investigated in other solid tumours is the use of fusion proteins comprised of a part recognising the tumour-specific antigen conjugated to a ligand for activating NK cell receptors, like NKG2D (Fig. 1b) [56]. However, these two strategies are of limited use in the highly heterogeneous brain tumours. There rarely exist in these malignancies tumour-specific antigens that are expressed on all the tumour cells in each patient and that are also absent on the normal cells. Moreover, it may be envisaged that these tumours would eventually escape the treatment by developing antigen loss variants. Reducing NK cell inhibition mediated by interactions of inhibitory KIR receptors with human leukocyte antigen (HLA) ligands may be achieved by blocking KIR receptors with specific antibodies (Fig. 1c). This strategy has been tested in multiple myeloma patients and proved to be safe [57]. Another alternative solution would be the use of allogeneic NK cells instead of patients’ own cells (Fig. 1d). The allogeneic NK cells have been shown to mediate the graft versus leukemia (GvL) effect in haematological malignancies [58]. The hypothesized mechanism is the receptor-ligand mismatch between the KIR receptors expressed on donor’s NK cells and cognate HLA ligands expressed by recipient’s cells resulting in the absence of inhibitory signals mediated by KIR receptors expressed by a subpopulation of NK cells [59]. Moreover, the patient’s normal cells are spared from killing due to the absence of activating ligands that are expressed almost exclusively on transformed cells. We hypothesise that the same strategy could be used in the treatment of solid tumours, including brain cancer. Moreover, a combination of autologous or allogeneic NK cell-based cellular therapy with immune stimulating treatment such as inhibitory KIR-blocking antibody could give better results compared to each of those strategies applied alone. Brain tumour patients could also benefit from combining adoptive transfer of NK cells with already approved therapeutic antibodies such as cetuximab, an anti-epidermal growth factor receptor (EGFR) antibody. Figure 2 summarizes the potential of combination approaches and the role of NK cells in anti-tumour immune response.

## Conclusions and perspectives: why do we need further investigation in this field?

The NK cells have limited access into the nascent brain [60] and they are the least abundant immune cell population within the brain tumour microenvironment. However, different methods used to detect NK cells in the tumour tissue led to conflicting results. Recent developments give an opportunity to study the infiltration of NK cells with more precise methods, such as with NK cell-specific antibodies or with multicolour flow cytometry with a panel of markers. It is also important to reveal the mechanism of NK cell recruitment, as various experimental therapeutic agents have been demonstrated to stimulate NK cell-mediated anti-tumour immune responses [8, 27, 28, 32]. NK cells might also influence the efficacy of other anti-cancer treatments [31], therefore their translation into clinic requires knowledge about the patients’ immunological status.

Malignant brain tumours develop multiple mechanisms of immune escape and both local, and systemic immunosuppression [33, 36, 45, 61, 62]. Many immunotherapies are based on patients’ own immune cells isolated from peripheral blood. Therefore, it is of great importance to investigate the functionality of autologous NK cells in brain tumour patients and the tumour-derived factors that could negatively influence NK cell-based therapy. So far, very few studies have been conducted and most of them investigated whole lymphocyte populations [33]. Research focused on pure NK cell fractions could facilitate the development of efficient treatment strategies directly utilizing NK cells as anti-cancer agents or stimulating endogenous NK cells.

To summarize, NK cells play an important role not only in anti-tumour immunity but they are potent effectors that may be considered for developing novel immunotherapies. However, stringent research is required to determine the impact of NK cell infiltration into the tumour site and their therapeutic efficacy in brain tumour patients. Investigating NK cells in the context of brain tumour is also essential for improving the NK cell-based immunotherapies that are increasingly investigated for clinical development for brain tumour patients.

## Abbreviations

ADCC: Antibody dependent cellular cytotoxicity
APC: Antigen presenting cell
BBB: Blood brain barrier
CpG-ODN: CpG-oligodeoxynucleotides
CTL: Cytotoxic T lymphocyte
DC: Dendritic cell
EGFR: Epidermal growth factor receptor
GBM: Glioblastoma
GvL: Graft versus leukemia (effect)
HLA: Human leukocyte antigen
IFN-γ: Interferon gamma
IHC: Immunohistochemistry
IL: Interleukin
KIR: Killer immunoglobulin-like receptors
LAK cells: Lymphokine activated killer cells
MHC: Major histocompatibility complex
MRI: Magnetic resonance imaging
NCR: Natural cytotoxicity receptor
NK cells: Natural killer cells
PBMCs: Peripheral blood mononuclear cells
rAAV2: Recombinant adeno-associated virus 2
SLMC: Spontaneous lymphocyte mediated cytotoxicity
TGFβ: Transforming growth factor-beta
TLR: Toll-like receptor
